# Emergency Medical Treatment and Labor Act (EMTALA) 2002-15: Review of Office of Inspector General Patient Dumping Settlements

**DOI:** 10.5811/westjem.2016.3.29705

**Published:** 2016-05-04

**Authors:** Nadia Zuabi, Larry D. Weiss, Mark I. Langdorf

**Affiliations:** * University of California Irvine, Department of Emergency Medicine, Irvine, California; † University of Maryland, Department of Emergency Medicine, Baltimore, Maryland

## Abstract

**Introduction:**

The Emergency Medical Treatment and Labor Act (EMTALA) of 1986 was enacted to prevent hospitals from “dumping” or refusing service to patients for financial reasons. The statute prohibits discrimination of emergency department (ED) patients for any reason. The Office of the Inspector General (OIG) of the Department of Health and Human Services enforces the statute. The objective of this study is to determine the scope, cost, frequency and most common allegations leading to monetary settlement against hospitals and physicians for patient dumping.

**Methods:**

Review of OIG investigation archives in May 2015, including cases settled from 2002–2015 ( https://oig.hhs.gov/fraud/enforcement/cmp/patient_dumping.asp ).

**Results:**

There were 192 settlements (14 per year average for 4000+ hospitals in the USA). Fines against hospitals and physicians totaled $6,357,000 (averages $33,435 and $25,625 respectively); 184/192 (95.8%, $6,152,000) settlements were against hospitals and eight against physicians ($205,000). Most common settlements were for failing to screen 144/192 (75%) and stabilize 82/192 (42.7%) for emergency medical conditions (EMC). There were 22 (11.5%) cases of inappropriate transfer and 22 (11.5%) more where the hospital failed to transfer. Hospitals failed to accept an appropriate transfer in 25 (13.0%) cases. Patients were turned away from hospitals for insurance/financial status in 30 (15.6%) cases. There were 13 (6.8%) violations for patients in active labor. In 12 (6.3%) cases, the on-call physician refused to see the patient, and in 28 (14.6%) cases the patient was inappropriately discharged. Although loss of Medicare/Medicaid funding is an additional possible penalty, there were no disclosures of exclusion of hospitals from federal funding. There were 6,035 CMS investigations during this time period, with 2,436 found to have merit as EMTALA violations (40.4%). However, only 192/6,035 (3.2%) actually resulted in OIG settlements. The proportion of CMS-certified EMTALA violations that resulted in OIG settlements was 7.9% (192/2,436).

**Conclusion:**

Of 192 hospital and physician settlements with the OIG from 2002–15, most were for failing to provide screening (75%) and stabilization (42%) to patients with EMCs. The reason for patient “dumping” was due to insurance or financial status in 15.6% of settlements. The vast majority of penalties were to hospitals (95% of cases and 97% of payments). Forty percent of investigations found EMTALA violations, but only 3% of investigations triggered fines.

## INTRODUCTION

The Emergency Medical Treatment and Active Labor Act (EMTALA) of 1986 was enacted to prevent discrimination of patients in hospital emergency departments (ED). During its debates and public hearings, Congress expressed its intent to ban financial discrimination and resultant “dumping” of uninsured patients on public hospitals. However, in its final legislated form, EMTALA bans discrimination of ED patients for any reason. It was enacted as part of the Consolidated Omnibus Budget Reconciliation Act (initially designated COBRA, 1985), 1 and modified as the Omnibus Budget Reconciliation Act (OBRA) of 1989. Increasing instances of refusal of care to patients with emergency medical conditions (EMC) prompted Congress to pass this unfunded mandate that required hospitals to:

1. Provide appropriate medical screening examinations (MSE) to the point of identifying or excluding an EMC
2. Stabilize EMCs according to the hospital’s capabilities
3. Provide timely consultation, treatment and hospitalization for the EMC within the “capacity” of the treating hospital and medical staff
4. Appropriately transfer unstable patients to a higher level of care (HLOC) if benefits outweigh the risks of transfer
5. Report known violations by hospitals and physicians receiving such transfers

In addition, the statute provided civil penalties for violation upon both hospitals and physicians. It is investigated by the Office of the Inspector General (OIG) of the Department of Health and Human Services’ (HHS) Centers for Medicaid and Medicare Services (CMS).^2^

EMTALA defines an EMC as either (1) a medical condition manifesting itself by acute symptoms of sufficient severity (including severe pain, psychiatric disturbances and/or symptoms of substance abuse) such that the absence of immediate medical attention could reasonably be expected to result in: placing the individual’s health (or, with respect to a pregnant woman, the health of the woman or her unborn child) in serious jeopardy; or serious impairment to bodily functions; or serious dysfunction of any bodily organ or part; or (2) with respect to a pregnant woman who is having contractions, that there is inadequate time to effect a safe transfer to another hospital before delivery, or that the transfer may pose a threat to the health or safety of the woman or the unborn child.^3^

Potential penalties for both hospitals and physicians are severe, despite the fact that neither is compensated for the cost of providing care to uninsured or underinsured patients. Physicians and hospitals are fined up to $50,000 per incident for failing to comply with EMTALA and are also at risk of exclusion from federally funded Medicare and Medicaid programs for repeated or flagrant violations.^1^ Furthermore, physicians and hospitals are liable regardless of intent, as determined in *Roberts v. Galen,* an EMTALA case that reached the U.S. Supreme Court in 1999.^4^

The regional offices of HHS and CMS are responsible for investigating complaints of alleged EMTALA violations and forwarding confirmed violations to the OIG for possible imposition of civil monetary fines.^2^ The regional CMS office usually delegates the initial onsite investigation to a state department of health.

Compliance with the EMTALA statute is difficult and costly, as every patient who has been deemed to have “come to the ED” must have an evaluation to exclude an EMC. Even the U.S. General Accounting Office (GAO) outlined some of these difficulties in a 2001 report. They include overcrowding from increasing ED patients with non-urgent complaints and reduced ED on-call specialist panels due to the inadequate compensation to care for a large number of uninsured patients. Furthermore, this report highlighted ambiguous regulations, including which satellite hospital sites fall under the EMTALA mandate.^2^

Despite 300 federal court decisions expanding interpretation of the statute with case law, the HHS maintains no ongoing transparent and public reporting system for potential violations. Even the U.S. GAO has criticized the OIG enforcement as inconsistent and weak.^5^ While the OIG publicly discloses settlements on its website, these have not been compiled or analyzed since 2006.^6^

This paper provides an update on the activity of EMTALA settlements reported by the OIG, as well as the scope, cost, frequency, regional location, and most common allegations leading to settlements by hospitals and physicians for EMTALA violations. The descriptions of these settlements uniformly include a statement that settlements were made without admission of guilt on the part of hospitals or physicians.

## METHODS

We reviewed the OIG EMTALA archives in May 2015, which included EMTALA cases settled from 2002–2015.^7^ (https://oig.hhs.gov/fraud/enforcement/cmp/patient_dumping.asp accessed May 2015 with most recent report 3/17/15.)

Each settlement includes a one-paragraph synopsis of the case, with varying detail. The website uniformly names the hospital, state, physician name (if applicable), and amount of fine. None mentioned loss of federal funding as a consequence. The description often included the general clinical diagnosis of the patient, mechanism of injury and age, and most importantly, category of alleged violation. Age was not uniformly reported, and at times the description included reference to multiple patients per report without ages.

Narrative descriptions were codified by one of the authors (NZ), and entered into an Excel spreadsheet (version 7.0, Microsoft Corporation, Redmond, WA, 2007). We recorded the following 12 categories of alleged violations:

1. Failure to screen for an EMC
2. Failure to stabilize a patient with an EMC
3. Inappropriate transfer of a patient with an EMC
4. Failure to transfer a patient with an EMC
5. Patient turned away for insurance or financial status
6. Patient in active labor
7. On-call physician refused to see patient with EMC
8. Patient with EMC inappropriately discharged
9. Hospital did not accept referral for transfer in of patient with EMC
10. No specialist physician available upon patient with EMC arrival
11. ED on ambulance diversion
12. Hospital where patient presented had capacity to care for EMC but refused

Each of the 192 entries in the OIG database was allocated to as many categories (1–12) as appropriate. Therefore, the number of allegations described here exceeds the number of OIG reports by design.

We obtained information regarding total number of EMTALA allegations (denominator) in the U.S. from three sources. The first was a website maintained by the Association of Healthcare Journalists (AHCJ).^8^ This database covers January 6, 2011, to May 13, 2015, (4.5 years). We used the search term 489.24, which is the CMS code designation for an allegation of EMTALA violation. We designate this as “AHCJ Website.”

Second, we also derived information on the total number of CMS EMTALA investigations from the AHCJ database from this same organization, which requires membership, and which we designate “AHCJ Database.”

Finally, we sought personal communication from the federal administrator designated “EMTALA Technical Lead - Hospital Program Analyst Survey & Certification Group – Division of Acute Care Services Centers for Medicare & Medicaid Services.”

Since this was an analysis of publicly available data, no human subjects’ approval was required.

## RESULTS

There were 192 settlement agreements (14 per year average for more than 4,000 hospitals in the U.S.). Fines against both hospitals and physicians totaled $6,357,000 (hospital and physician average $33,435 and $25,625 respectively). There were 184 (95.8%, $6,152,000) settlements involving hospitals and eight (4.2%) against physicians ($205,000). Therefore, 97% of monetary penalties were levied against hospitals.

**Figure 1:**
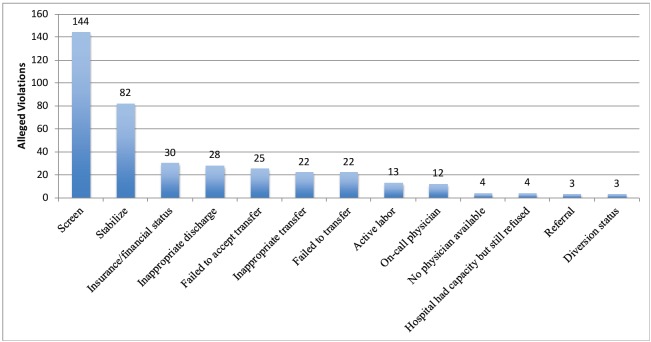
Factors associated with monetary settlement with U. S. Office of Inspector General for allegations of violation of Emergency Medical Treatment and Labor Act (EMTALA), 2002–15 (n= 392 for all categories of violations).

There were 392 alleged categories (1–12) of EMTALA violations identified in these 192 brief reports from the OIG website (average 2.04 per settlement). The categories of settlements are detailed in Figure 1. The most common settlements were for failing to screen (144/192, 75%) and stabilize (82/192, 42.7%) for EMCs. Other factors are listed in Figure 1.

Although loss of Medicare/Medicaid federal funding is an additional possible penalty for EMTALA violation, there were no reported cases where hospitals were excluded from federal reimbursement. There was no information on EMTALA investigations that were not subject to settlement.

**Figure 2:**
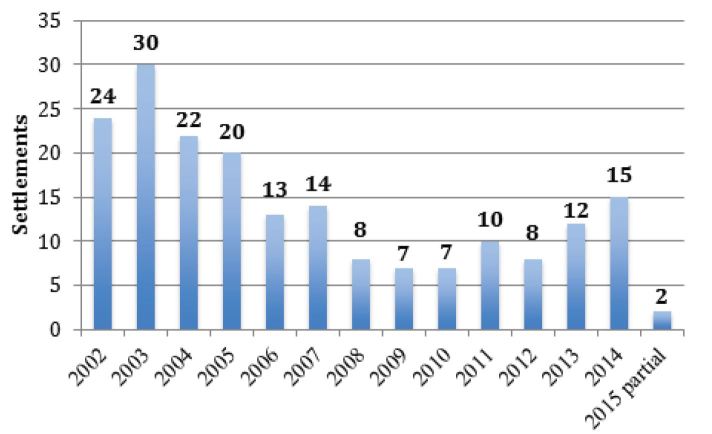
Number of monetary settlements with the U.S. Office of Inspector General for allegations of violation of Emergency Medical Treatment and Labor Act (EMTALA), 2002–15 (n=192).

The number of settlements per year varied during the study period (Figure 2), from a high of 30 in 2003 to lows of seven per year in 2009 and 2010 (2015 is a partial year).

We sought to determine the proportion of EMTALA investigations that resulted in fines, requiring a denominator of EMTALA investigations from 2002–15. This was derived from three sources, which yielded different results.

The AHCJ Website (found at www.Hospitalinspections.org ) returned 359 records of hospitals investigated from 2011–15. We scrutinized each to assure there was indeed at least one EMTALA-based allegation recorded, including the categories above, and eliminated duplicates. This yielded 338 unique EMTALA allegations in 4.5 years. Earlier data were not compiled from this source.

The AHCJ Database downloaded directly from the subscription to the organization listed 527 instances of “investigation for violation of EMTALA” between January 2011 and January 2015 (4 years), among 14,516 total CMS hospital offenses that led to investigations for all causes (3.6%).

**Figure 3:**
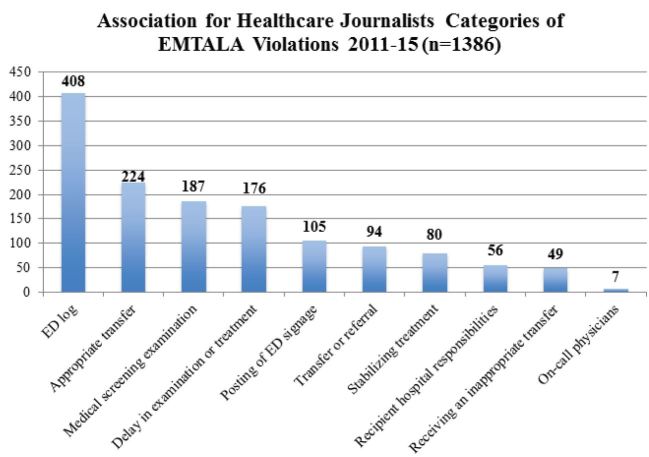
Emergency Medical Treatment and Labor Act (EMTALA) violations (2011–15) by category, from the Association of Healthcare Journalists Database. These 1,386 categories of alleged violations were contained in 527 separate CMS (Centers for Medicare and Medicaid Services) investigations. *ED,* emergency department

Of the 14,516 offenses listed in the AHCJ Database that were investigated by CMS, 10 specific EMTALA violation categories were listed. These were not the same as the 12 categories of violations identified by the authors of this paper, which were contained in the OIG narrative descriptions. For example, the authors of this paper identified four additional categories of violations not in the AHCJ list: 1) active labor, 2) turned away for financial status, 3) no specialty physician available, and 4) ED on ambulance diversion. Conversely, the AHCJ Database had two categories that the authors did not find in the OIG website: 1) ED log (a required list of all patients who present to any ED in chronological order) and 2) posting of signage regarding EMTALA obligations. The sum of all allegations in the AHCJ Database was 1,386 (multiple allegations in each of 527 investigations), and we report the breakdown of these alleged violations by category in Figure 3.

Therefore, depending on the denominator taken from the AHCJ Database (n=527) or the AHCJ Website (n=338), there were between approximately 75–130 EMTALA investigations per year from 2011–2015. Extrapolating these 4 to 4.5 years of data over the 12-year period of OIG listed EMTALA settlements would amount to approximately 100 per year, or 1,200 investigations. Given 192 monetary settlements, this indicates that, at most, approximately 16% of EMTALA investigations result in fines.

The third source of information (and perhaps most reliable) on the scope of EMTALA allegations and investigations is CMS’ own database, operational since 2004. Between then and 2015, there were 6,316 complaints received (approximately 574 per year), 6,035 investigations done by CMS (549/year), of which 2,436 found EMTALA violations (221/year).^9^ This source documents more than four times as many investigations per year as the AHCJ website or database contain. Using these data to extrapolate over the 12-year period during which OIG settlements are publicly reported, would drop the proportion of investigations that result in OIG fines to 3.2% (192/6,035). Furthermore, the proportion of even CMS findings of liability (n=2,436) that resulted in OIG settlements occurred in only 7.9% (192/2,436) of cases.

**Figure 4:**
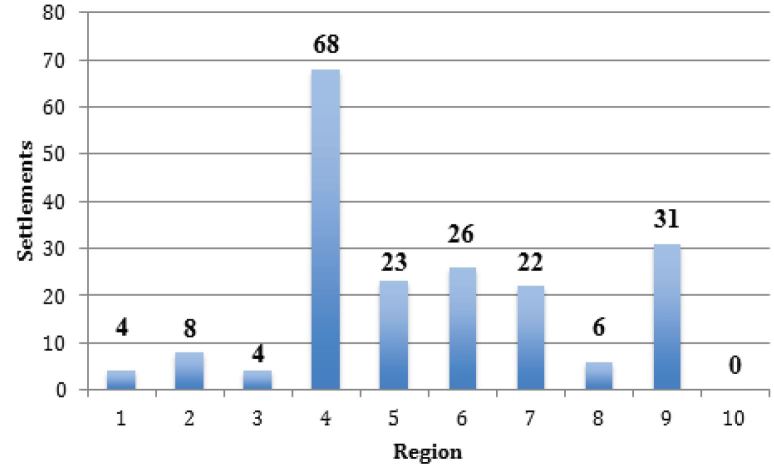
Number of monetary settlements with U.S. Office of Inspector General by Centers for Medicare and Medicaid (CMS) region, for Emergency Medical Treatment and Labor Act (EMTALA) violations, 2002–15.

**Figure 5:**
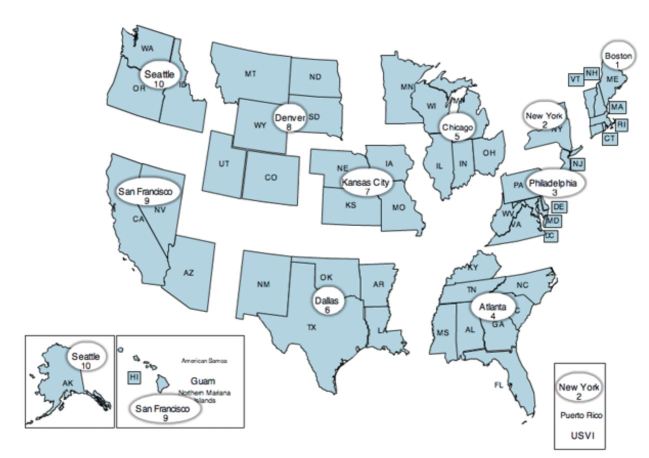
Center for Medicare and Medicaid (CMS) regions relevant to reporting of U.S. Office of Inspector General Emergency Medical Treatment and Labor Act (EMTALA) enforcement.

There was significant variation in the number of settlements by CMS region (Figure 4), from a high of 68 in Region 4 (Southeast) to a low of 0 in Region 10 (Pacific Northwest), shown in Figure 5.

## DISCUSSION

This paper adds unique data to the emergency medicine literature, as it provides the first review of recent CMS EMTALA investigations and civil monetary penalties. As the investigation process is complaint-driven, these data reveal that most complaints relate to allegations of improper screening examinations, followed by improper stabilization, and then improper transfers, but only a minority of allegations result in fines.

The EMTALA mandate began in 1986 in response to high-profile cases where patients were denied care, or were transferred from the ED without receiving necessary stabilizing care. Prior to EMTALA, in many communities the under- and uninsured received care in public hospitals (usually academic medical centers), while privately insured patients often received care in privately-owned community hospitals. When patients without insurance presented with medical emergencies or active labor to private hospitals, they were often turned away, some with bad outcomes in transit, or after arrival at public hospitals.

Rather than dealing directly with inequitable funding of providers and hospitals for healthcare, EMTALA placed an unfunded mandate on EDs and on-call specialists to provide screening and stabilizing care to everyone, without regard for insurance or ability to pay.

Consequently, regional surveys of community hospital EDs in California documented the erosion of on-call specialty panels from 2000 to 2006. The EMTALA mandate was an important driver, as specialists refused to take ED call to avoid being subjected to EMTALA mandates to care for the unfunded.^10^ This further shifted underfunded and unfunded care to university and public hospitals.

This trend accelerated after November 2003 when CMS significantly amended the EMTALA regulations. The new amendments significantly changed the duty of hospitals to provide panels of on-call physicians to their EDs. Prior to that time, the on-call panel had to include all specialties represented in the organized medical staff of the hospital. After November 2003, hospitals only had to provide an on-call panel that reasonably met the needs of its community.^11^ Very quickly, surgical subspecialists began disappearing from hospital call schedules. The “needs of the community” were then served by transferring patients to academic medical centers and other tertiary care facilities. Therefore, for a variety of reasons, the post-EMTALA provision of emergency care no longer involves many specialists in some communities.

However, during this time, the number of settlements appears to have decreased. After 30 years of implementation, a whole generation of physicians has been exposed to safer evaluation of potential medical emergencies prior to consideration for transfer, and to the prohibition of inquiry regarding method of payment prior to MSE.

To further understand this, it is instructive to examine the CMS’s “EMTALA Physician Review Worksheet,” which gives good insight into the factors involved in an investigation (Appendix). First, there are instructions defining the medical screening examination, which can range from a “brief history and physical examination,” to a “complex process” involving ancillary studies, diagnostic procedures (such as lumbar puncture), advanced imaging, consultation and procedures performed by consultants.

The standard asked of the CMS physician reviewer is one of “reasonable clinical confidence,” sufficient to determine whether or not an EMC…existed.” The form asks the reviewer to identify “inappropriately long delay” prior to the MSE. “In labor” is defined as “having contractions,” without further specifying interval or severity. The reviewer is asked to determine, “with reasonable medical certainty” whether there would be time to transfer the pregnant patient safely. Patient outcome after transfer is noted to not be a determinant of appropriate stabilization, but this could be a “red flag.” However, regarding transfer of a woman in labor, delivery prior to arrival at the receiving hospital is considered a “marker of instability” for transfer.

The obligation for care includes that which is “within the full capabilities” of a hospital’s staff and facilities and includes access to on-call specialists. The form asks the investigator to state the reason the EMC was not stabilized prior to transfer, and notes that the patient can refuse stabilizing treatment. During the transfer, the form asks whether this was done with the best equipment and personnel, whether the benefits outweighed the risks of transfer, whether the physician documented the benefits and risks in the medical record, and if the patient was sent with all medical records.

Designation as a specialty center is not limited to, for example, trauma/burn/neonatal intensive care unit (ICU), but rather asks the investigator to opine on other hospital capabilities. On the receiving end, hospital’s ability to accept a transfer can be limited by “lack of capacity,” but this is not further defined. Disputes arise whether a receiving hospital can refuse a transfer because of lack of inpatient, ICU or operating rooms, in addition to ED beds. This element is vague in all EMTALA guidance from CMS and the statute itself. The investigator worksheet similarly leaves this undefined.

The worksheet instructs the investigator specifically to *not* render an opinion regarding EMTALA violation. Finally, it requires the listing of the specialty of any potential violator physician, implying this is not limited to the actions of the emergency physician, but includes on-call specialists as well.

There is a small chance that any individual case will lead to an EMTALA investigation and fine. However, this could have devastating consequences for a physician or hospital. A $50,000 fine may have a minimal impact for a hospital, but a significant impact on a physician, as malpractice insurance policies do not cover the cost of civil penalties. Also, when a regional CMS office authorizes an on-site investigation, they can also investigate other previous cases involving the hospital and the physician. If CMS finds repeated or flagrant violations, each violation may result in an additional fine and may result in permanent exclusion of the providers from the Medicare and Medicaid programs.

Even though CMS may fine both hospitals and physicians for EMTALA violations, a patient may only legally sue the hospital for alleged injuries due to an EMTALA violation. Even though physicians do not face tort liability for alleged EMTALA violations, the duties enumerated by EMTALA quickly became national standards of practice. Therefore, in medical malpractice litigation, patients may allege negligence due to failure of a physician to adhere to national standards of practice, including screening, stabilization, and transfer of ED patients.

A discussion of alleged EMTALA violations must include the three major obligations of hospitals and physicians, the duties to appropriately screen, stabilize, and transfer patients. Congress failed to provide a definition of appropriate MSE, and this led to more litigation than any other aspect of the statute. Gradually, the federal courts of appeals developed the “comparability test.” A hospital provides an appropriate MSE when it provides an examination comparable to a similar patient.^12^ This reflects the fact that EMTALA is an anti-discrimination statute.

If the MSE does not reveal an EMC, then EMTALA obligations are fulfilled. However, the diagnosis of an EMC gives rise to the duty to stabilize.^12^,^13^ A patient is stable if it is reasonably likely the patient will not deteriorate en route during a transfer, including patients “transferred” to home (discharged from the ED).^14^ When providers stabilize the patient’s EMCs the obligations under EMTALA are fulfilled.^14^,^15^

The EMTALA transfer obligations only apply to unstable patients. Therefore, if an ED cannot stabilize a patient, then it may transfer the patient if “appropriate.”^16^ The referring hospital must stabilize the patient to its maximum potential, must secure acceptance from a provider at a receiving hospital, and send appropriate records, personnel and equipment. The referring physician must sign a certification, in actuality an oath, documenting that the medical benefits of transfer outweigh the risks. In addition to these “appropriate” transfer requirements, patients may request or demand transfer after proper disclosure of the risks.

A receiving hospital has a duty to accept all “appropriate” transfers if it has capacity (an available appropriate bed) and the capability (appropriate staff), and if the receiving hospital serves as a regional referral center or has unique capabilities not available at the referring hospital.^16^ Hospitals do not have a duty to accept lateral transfers.^17^

We were not able to find any recent hospital or physician loss of federal funding in our investigation. The last information from 2007 from CMS reported 13 hospitals had been terminated from Medicare for EMTALA violations.^6^

We had substantial difficulty discovering the true number of EMTALA allegations that may result in penalties. We believe the federal government’s CMS data to be most accurate, suggesting a very low (<8%) incidence of OIG monetary penalties even after CMS determines there has been an EMTALA violation.^9^

## CONCLUSION

Of 192 hospitals and physicians settling with the OIG from 2002–15, most were for failing to provide screening (75%) and stabilization (42%) to patients with EMCs. The reason for patient “dumping” was due to insurance or financial status in 15.6% of settlements, the original intent of the statute. The vast majority of penalties were to hospitals (95% of cases and 97% of fines). Forty percent of investigations found EMTALA violations, but only 3% of investigations triggered fines.

## Supplementary Information
