# Supplementary material for: Emergency Medical Treatment and Labor Act (EMTALA) 2002-15: Review of Office of Inspector General Patient Dumping Settlements
Source: West J Emerg Med. 2016 May 4;17(3):245–51. doi: 10.5811/westjem.2016.3.29705 (PMC4899053; doi:10.5811/westjem.2016.3.29705)
Supplement: Supplementary file 1 [file wjem-17-245-s001.pdf]

EXHIBIT 138

(Rev. 134, Issued: 02-20-15, Effective: 02-13-15, Implementation: 02-13-15)

EMTALA Physician Review Worksheet

(●) **5 - Day Review** ( ) **60 - Day Review**

NOTE: *A separate Worksheet must be completed by the QIO Physician Reviewer for each medical record reviewed. To facilitate accurate completion, the CMS Regional Office (RO) will complete Section I for each medical record sent to the QIO along with the request for review. The RO must label each medical record with the unique patient identifier as found on the draft Form CMS 2567.*

SECTION I

Complaint Control Number: CA00442282 Patient *Identifier Number on Draft 2567*: #31

Name of Patient: ________________ DOB: ________________

Name of Alleged Violating Hospital and/or Physician: Seton Medical Center/Seton Coastside

City: Daly City/Moss Beach State: CA CMS Certification Number: 050289

Date and Time of Admission to Emergency Services: 9/1/2014@12:11 p.r

Date and Time of Discharge from Emergency Services: 9/1/2014@14:09 p.r

Name of Receiving Hospital (if applicable): ________________

Receiving Hospital Location:

City: ________________ State: ________________ CMS Certification Number: ________________

Date and Time of Admission to Receiving Hospital (if applicable): ________________

Manner of Transport: ________________

Receiving Hospital Distance from Sending Hospital (if applicable and known): ________________

SECTION II

Note to Physician Reviewer: Please complete the following questions to address issues related to EMTALA. Please be sure to include your clinical rationale for your *findings*, and make any summary comments and comments on other aspects of the case in the summary section on the last page of this document. Please keep in mind that the purpose of your comments is to provide your clinical perspective on the care rendered, for the CMS 5-day EMTALA review or for the OIG 60-day EMTALA review.

Therefore, please refrain from making ANY statements about whether or not a violation of EMTALA has occurred, as that decision is the responsibility of CMS and the OIG only.

(Violations of EMTALA may also constitute negligence under state malpractice law. *However, determining negligence is not part of and should not be mentioned in your EMTALA review*.)

EXHIBIT 138

(Rev. 134, Issued: 02-20-15, Effective: 02-13-15, Implementation: 02-13-15)

EMTALA Physician Review Worksheet

MEDICAL SCREENING EXAMINATION

Note to Physician Reviewer: Depending upon an individual's presenting symptoms, an appropriate medical screening examination can range from a simple process involving only a brief history and physical examination to a complex process that also involves performing ancillary studies and procedures such as (but not limited to) lumbar puncture, clinical laboratory tests, CT scans and other diagnostic tests and procedures, *some of which may require the services of an on-call specialist to order, conduct or interpret.*

A hospital must provide appropriate screening services within the full capabilities of its staff and facilities, including access to specialists who are on call.

An **Emergency Medical Condition** is defined as **EITHER**: (1) a medical condition manifesting itself by acute symptoms of sufficient severity (including severe pain, psychiatric disturbances and/or symptoms of substance abuse) such that the absence of immediate medical attention could reasonably be expected to result in: placing the individual's health (or, with respect to a pregnant woman, the health of the woman or her unborn child) in serious jeopardy; or serious impairment to bodily functions; or serious dysfunction of any bodily organ or part; **OR** (2) with respect to a pregnant woman who is having contractions, that there is inadequate time to effect a safe transfer to another hospital before delivery, or that the transfer may pose a threat to the health or safety of the woman or the unborn child. *(See 42 CFR 489.24(b))*

1. Did the hospital provide a medical screening examination that was, *within reasonable clinical confidence, sufficient to determine whether or not an EMERGENCY MEDICAL CONDITION (as defined above) existed? More specifically:*

1a. ***Was the medical screening examination appropriate given all of*** **the individual's medical complaints and signs and symptoms** ***at the time the individual presented*****?**

○ **YES** ○ **NO**

Please explain your clinical rationale:

EXHIBIT 138

(Rev. 134, Issued: 02-20-15, Effective: 02-13-15, Implementation: 02-13-15)

EMTALA Physician Review Worksheet

1b. ***Was the medical screening examination appropriate given the hospital's capabilities*** **- including ancillary services routinely available and consultations by on–call specialist physicians?**

◯ **YES** ◯ **NO**

Please explain your clinical rationale:

1c. Is there any evidence that there was an inappropriately long delay, based on the individual's clinical presentation, between the individual's arrival and the provision of an appropriate medical screening examination?

◯ **YES** ◯ **NO**

Please explain your clinical rationale:

EXHIBIT 138

(Rev. 134, Issued: 02-20-15, Effective: 02-13-15, Implementation: 02-13-15)

EMTALA Physician Review Worksheet

EMERGENCY MEDICAL CONDITION

2. Did this individual have an EMERGENCY MEDICAL CONDITION as defined by Part (1) of the definition noted above? (Individual conditions meeting the definition in Part 2 above are addressed in subsequent questions.)

◯ **YES** ◯ **NO**

Please explain your clinical rationale:

3. Was this individual a pregnant woman who was having contractions?

◯ **YES** ◯ **NO**

Please explain your clinical rationale:

(If "NO" is checked, skip questions #3a & #3b and proceed to #4)

EXHIBIT 138

(Rev. 134, Issued: 02-20-15, Effective: 02-13-15, Implementation: 02-13-15)

EMTALA Physician Review Worksheet

3a. If "YES" *is checked in #3* and the pregnant woman was transferred/discharged, at the time of transfer/discharge, could it be determined with reasonable medical certainty that there would be adequate time to effect a safe transfer to another hospital before delivery?

○ **YES** ○ **NO** ○ **N/A**

Please explain your clinical rationale:

3b. If *"YES" is checked in #3 and* the pregnant woman with contractions was transferred/discharged, at the time of transfer/discharge could it be determined, with reasonable medical certainty, that the transfer/discharge would not pose a threat to the health or safety of the *pregnant woman* or the unborn child?

○ **YES** ○ **NO** ○ **N/A**

Please explain your clinical rationale:

EXHIBIT 138

(Rev. 134, Issued: 02-20-15, Effective: 02-13-15, Implementation: 02-13-15)

EMTALA Physician Review Worksheet

STABILIZING TREATMENT

Note to Physician Reviewer: Terms relating to "stabilization" are specifically defined under EMTALA. These terms DO NOT REFLECT the common usage in the medical profession, but instead focus on the medical risks associated with a particular transfer/discharge. Thus, when answering questions related to "stability" for EMTALA, please be very careful to refer to the definition provided below. In addition, the clinical outcome of an individual's condition is not a proper basis for determining whether a person transferred was stabilized. However, the individual's outcome may be a "red flag" indicating that a more thorough evaluation of the individual's condition at the time of transfer was needed.

*Under EMTALA, **to stabilize** means, with respect to part 1 of the definition of an "emergency medical condition," to provide such medical treatment of the condition necessary to assure,* within reasonable medical probability, that no material deterioration of the condition is likely to result from or occur during the transfer/discharge of the individual from the hospital, or in the case of *part 2 of the definition, concerning* a pregnant woman having contractions, that the pregnant woman has delivered the child and placenta.

4. If *the individual had* an emergency medical condition (EMC), was the EMC "stabilized" *(as defined above) prior to the time of the individual's transfer or discharge?*

◯ **YES** ◯ **NO** ◯ **N/A**

Please explain your clinical rationale:

EXHIBIT 138

(Rev. 134, Issued: 02-20-15, Effective: 02-13-15, Implementation: 02-13-15)

EMTALA Physician Review Worksheet

Note to Physician Reviewer: *A hospital must provide appropriate stabilizing treatment services for an emergency medical condition within the full capabilities of its staff and facilities, including access to specialists who are on call.*

5a. Is there any evidence that the hospital was equipped with such staff, services, or equipment necessary to "stabilize" the individual's emergency medical condition??

○ **YES** ○ **NO** ○ **N/A**

Please explain your clinical rationale:

5b. If the hospital had the capability to stabilize the individual and the individual's *emergency medical condition* was not stabilized prior to transfer/discharge, is there any information available to indicate WHY the emergency medical condition was NOT "stabilized" prior to discharge/transfer?

○ **YES** ○ **NO** ○ **N/A**

If yes, does this rationale have a sound clinical basis?:

EXHIBIT 138

(Rev. 134, Issued: 02-20-15, Effective: 02-13-15, Implementation: 02-13-15)

EMTALA Physician Review Worksheet

5c. Is there any evidence that there was an inappropriately long delay, based on the individual's clinical presentation, between the individual's arrival and the provision of appropriate stabilizing treatment for the individual's emergency medical condition?

◯ ***YES*** ◯ ***NO*** ◯ ***N/A***

Please explain your clinical rationale:

Note to Physician Reviewer: *A hospital is required to inform the individual or the individual's legal representative of the risks and benefits of further examination and treatment. If the individual/representative then refuses to consent to further examination or treatment, the medical record must contain a description of the examination or treatment, or both, which was refused, as well as documentation of the individual/representative having been informed of these risks/benefits.*

6. Does the medical record indicate the individual refused to consent to necessary stabilizing treatment?

◯ ***YES*** ◯ ***NO***

(If "NO" is checked, skip question #6a and proceed to #7)

6a. If "YES" is checked and if the medical record contains a description of the communication to the individual/legal representative of the risks and benefits and benefits of further examination or treatment, was this communication appropriate, based on the information available to the hospital at the time of the refusal?

◯ ***YES*** ◯ ***NO*** ◯ ***N/A***

Please explain:

EXHIBIT 138

(Rev. 134, Issued: 02-20-15, Effective: 02-13-15, Implementation: 02-13-15)

EMTALA Physician Review Worksheet

APPROPRIATE TRANSFERS

7a. If your response to question 5a was "NO" finding that the hospital was not capable of stabilizing the individual's emergency medical condition, **what were the required specialized capabilities that the hospital lacked?**

7b. **If the individual was transferred to another hospital, did the** ***sending*** **hospital provide further examination and stabilizing treatment, within its** *capacity* **(including ancillary services routinely available to it) to minimize the risks of transfer to the individual's health and, where relevant, the health of the unborn child?**

◯ **YES** ◯ **NO** ◯ **N/A**

Please explain your clinical rationale:

EXHIBIT 138

(Rev. 134, Issued: 02-20-15, Effective: 02-13-15, Implementation: 02-13-15)

EMTALA Physician Review Worksheet

8. If the individual was transferred to another hospital, to minimize the risks of transfer, *were* qualified personnel and transportation equipment, including *medically appropriate* life support measures, *used to effect (i.e., accomplish) the transfer*?

◯ **YES** ◯ **NO** ◯ **N/A**

Please explain your clinical rationale:

9a. ***If this individual was transferred to another hospital for stabilizing treatment of an unstabilized emergency medical condition, do you find that, considering the individual's clinical condition at the time of transfer and any other pertinent information available at that point in time, the medical benefits reasonably expected from appropriate medical treatment at the other hospital outweighed the increased risk to the individual (or woman in labor or unborn child) from being transferred?***

◯ **YES** ◯ **NO** ◯ **N/A**

Please explain your clinical rationale:

EXHIBIT 138

(Rev. 134, Issued: 02-20-15, Effective: 02-13-15, Implementation: 02-13-15)

EMTALA Physician Review Worksheet

Note to physician reviewer: *The physician certification required for an appropriate transfer must be in writing, must contain a summary of the specific risks and benefits pertaining to this individual's clinical situation, and must be placed in the individual's medical record.*

9b. ***Do you find that the summary of risks and benefits of transfer contained in the physician certification was appropriate, based on the information available to the hospital at the time of transfer about the individual's condition?***

○ **YES** ○ **NO** ○ **N/A***

Please explain:

**Check N/A not only if this case does not involve a transfer, but also if there was no physician certification in the medical record*

9c. ***If the transfer was at the request of the individual or the individual's legal representative, rather than based on a physician's certification of the benefits outweighing the risks, and the medical record documents this, do you find that the likely risks of the transfer were identified for the individual/representative?***

○ **YES** ○ **NO**

Please explain your clinical rationale:

EXHIBIT 138

(Rev. 134, Issued: 02-20-15, Effective: 02-13-15, Implementation: 02-13-15)

EMTALA Physician Review Worksheet

10. **Does the documentation suggest that the transferring hospital sent to the receiving hospital all available and pertinent medical documentation related to the emergency medical condition?**

○ **YES** ○ **NO** ○ **N/A**

Please explain:

RESPONSIBILITY OF HOSPITALS WITH SPECIALIZED DIAGNOSTIC OR TREATMENT CAPABILITIES OR FACILITIES

Note to Physician Reviewer: While "specialized capabilities or facilities" include such facilities as burn units, shock-trauma units, neonatal intensive care units or regional referral centers, it also includes *many more clinical characteristics*. Most simply, if an individual with an emergency medical condition needs services to stabilize that condition that cannot be made available in a clinically appropriate timeframe at the hospital where the individual presented, but which are available at another hospital, the hospital with these capabilities/services must accept a request for transfer, if it has the capacity to provide the needed *stabilizing treatment*.

11. **Is there any evidence that a Medicare-participating hospital that refused a transfer request has specialized capabilities or services (not available at the *sending* hospital) that the individual required?**

○ **YES** ○ **NO** ○ **N/A**

Please explain:

(If "NO" or "N/A" is checked, skip question #11a and go to #12.)

EXHIBIT 138

(Rev. 134, Issued: 02-20-15, Effective: 02-13-15, Implementation: 02-13-15)

EMTALA Physician Review Worksheet

11.a If "YES" is checked in #11, is there evidence that the hospital with specialized capabilities or services lacked the capacity to treat the individual requesting stabilizing treatment, at the time of the request?

Please explain:

QUALITY

12. **Do you have any specific concerns about the quality of care rendered to the individual that have not already been addressed fully above?**

◯ **YES** ◯ **NO**

If yes, please explain your clinical rationale:

EXHIBIT 138

(Rev. 134, Issued: 02-20-15, Effective: 02-13-15, Implementation: 02-13-15)

EMTALA Physician Review Worksheet

SUMMARY OF FINDINGS:

13. Please summarize the key facts of the case below and any concerns or clarifications to your answers above with regard to this case. Remember, do not state an opinion regarding whether EMTALA was violated.

EXHIBIT 138

(Rev. 134, Issued: 02-20-15, Effective: 02-13-15, Implementation: 02-13-15)

EMTALA Physician Review Worksheet

I agree to provide medical advice to the Centers for Medicare & Medicaid Services and/or the Office of Inspector General, as necessary, to properly adjudicate any issues and to testify as an expert witness on behalf of the Office of Inspector General, if necessary.

Physician Reviewer Name (printed): ____

Physician Reviewer Signature: ____

Specialty: _______________________________________ Date: ________________________

***Case ID*:** ___________________________________

Time Required to Complete This Review: ________________hours ____________minutes
